# Molecular analysis of foveoschisis in females reveals a novel case of segmental uniparental disomy in X-linked retinoschisis

**DOI:** 10.1007/s10633-025-10053-y

**Published:** 2025-10-22

**Authors:** Nagham Maher Elbagoury, Mona Lotfi Essawi, Heba Mahmoud Fathy, Ola Mohamed Eid, Mostafa Nabih, Amal Mahmoud Mohamed, Caroline Atef Tawfik

**Affiliations:** 1 Department of Medical Molecular Genetics, National Research Centre, Cairo, Egypt; 2 Department of Human Cytogenetics, National Research Centre, Cairo, Egypt; 3 Department of Ophthalmology, Faculty of Medicine, Cairo University, Cairo, Egypt; 4 Department of Ophthalmology, Faculty of Medicine, Ain Shams University, 38 Abbasseya, Nour Mosque, El-Mohamady, Al-Waili, Cairo, 11566 Egypt

**Keywords:** Foveoschisis, X-linked retinoschisis, Stellate nonhereditary idiopathic foveomacular retinoschisis, *RS1* gene, Uniparental isodisomy, SNP array

## Abstract

**Background:**

Foveoschisis refers to the splitting of retinal layers involving the macula that may have different causes with variable structural–functional natural histories. Idiopathic cases are seen in the absence of inherited or acquired predisposing conditions and referred to as stellate nonhereditary idiopathic foveomacular retinoschisis (SNIFR). Our study aimed to clinically and genetically characterize females presenting with foveoschisis (including affected male siblings where present).

**Methods:**

Five patients (3 females and 2 males) from 3 consanguineous families presenting with foveoschisis underwent complete ophthalmological evaluation, multimodal imaging including color, infrared, fundus autofluorescence (FAF), spectral-domain optical coherence tomography (SD-OCT), electroretinogram (ERG), and molecular evaluation including Sanger sequencing of the *RS1* gene and whole exome sequencing (WES). Main outcome measures were age at first visit, best-corrected visual acuity (BCVA), peripheral retinal changes, FAF pattern, ERG findings, and *RS1* variants.

**Results:**

The mean age was 21.8 years. The BCVA ranged from 20/100 to 20/20. Peripheral retinal changes ranged from a tapetal reflex, peripheral retinoschisis, vitreous veils, to vitreoretinal traction. A ring of increased signal was the most common FAF abnormality, while one patient exhibited a double-ring hyperautofluorescence. All patients demonstrated an electronegative ERG. One female was considered to have a molecularly undiagnosed inherited retinal disease (IRD). Another female was considered stellate nonhereditary idiopathic foveomacular retinoschisis (SNIFR) after exclusion of other causes. Three patients showed a novel nonsense variant in the *RS1* gene; homozygous in the female sibling and hemizygous in the male siblings. Familial segregation revealed an unaffected father and a carrier mother. Trio SNP array confirmed maternal segmental uniparental isodisomy (seg UPiD).

**Conclusion:**

This is the first reported X-linked retinoschisis (XLRS) case with seg UPiD. We emphasize the significance of SNP arrays in elucidating non-Mendelian inheritance cases. We report a novel variant, which is the first to be detected in the RS1 domain in a female.

**Supplementary Information:**

The online version contains supplementary material available at 10.1007/s10633-025-10053-y.

## Introduction

Foveoschisis refers to the splitting of retinal layers involving the macula. It can have various underlying causes, including X-linked retinoschisis (XLRS) [1], myopic traction maculopathy [2–4], optic disc pit maculopathy [5–7], glaucoma [8, 9], vitreomacular traction (VMT) [10], enhanced S-cone syndrome (ESCS) [11, 12], *CRB1*-associated retinopathy [13, 14], myotonic dystrophy [15], and drug-induced by either niacin, a vitamin B complex derivative, or taxanes, which are chemotherapy agents [16]. Idiopathic cases seen in the absence of inherited or acquired predisposing conditions are referred to as stellate nonhereditary idiopathic foveomacular retinoschisis (SNIFR) [17]. Clinically, these conditions all present with similar phenotypes; however, they often exhibit variable structural–functional natural histories.

XLRS (OMIM #312,700) is an X-linked recessive vitreoretinal disease. It is the principal cause of degeneration of the macula in males [18]. Despite being almost exclusive in males, few studies reported female patients diagnosed either solely on clinical grounds or on both clinical and molecular grounds [19]. The estimated prevalence of XLRS ranges from 1:5000 to 1:25,000 [20]. The clinical diagnosis is based on retinal splitting near the outer plexiform layer (OPL), with peripheral schitic cavities observed in more than half of cases, mostly involving the inferotemporal quadrant. Less common peripheral changes include vitreous veils, perivascular sheathing, peripheral dendriform lesions, diffuse white retinal flecks, and Coat disease-like exudative retinopathy. The electroretinogram (ERG) is characteristically electronegative, with a reduction in the b-wave amplitude [18, 21, 22].

XLRS manifests due to retinoschisin protein deficiency. Retinoschisin regulates fluid balance between the intracellular and extracellular environment, particularly within the photoreceptor and bipolar cell layers [1]. Additionally, retinoschisin plays a role in cell adhesion, which is necessary for the development and maintenance of the retinal architecture. The most crucial role played is during retinal development immediately after neuronal birth and the differentiation of neuronal cells, which are maintained throughout life [18].

The protein retinoschisin is encoded by the *RS1* gene (OMIM# 312,700), which is located on the X chromosome at position Xp22.1. This gene is composed of six exons and spans approximately 14 kb. Retinoschisin is a 224-amino acid protein with four distinct regions: a 23-amino acid N-terminal leader or signal sequence (SS), a 39-amino acid RS1 domain, a 157-amino acid discoidin domain, and a 5-amino acid C-terminal segment (Ct) [1, 23]. The N-terminal SS is a cleavable signal sequence at position 23, which generates a 201-amino acid mature protein and facilitates its transport into the endoplasmic reticulum (ER) via the secretory pathway [24]. The RS1 domain and the C-terminal segment are crucial for oligomerization of retinoschisin, while the discoidin domain is essential for cell adhesion [25].

Precise clinical and molecular diagnosis of inherited retinal diseases (IRDs) and XLRS in particular can help proper management of the condition and genetic counseling. Differentiating the cause will clarify the risk of transmission to offspring, refine the follow-up schedule, and facilitate monitoring for potential complications, with accurate patient education. With about 25 studies targeting the *RS1* gene [20], and recent reports of rescue following gene augmentation therapy in retinal organoids with (p.E72K) variant [26], a precise genetic diagnosis is essential to gain access to emerging gene therapy trials. Our study aimed to determine the exact etiology of foveoschisis and characterize its clinical and molecular findings.

## Methods

Clinical records of three female patients presenting with foveoschisis to the outpatient clinics of Ain Shams and Cairo University between September 2018 and September 2022 were reviewed. All male relatives were examined, and two affected male siblings of one female were further recruited. Written informed consent was signed by the patients or their guardians after an explanation of the purpose of the study.

The study adhered to the tenets of the Declaration of Helsinki. Ethical approval was obtained from the Institutional Review Board of Research Ethics Committee at the Faculty of Medicine, Ain Shams University (FMASU R231/2024).

### Clinical evaluation

All patients underwent complete ophthalmological examination, including recording of medical, ocular, and family histories, unaided and best corrected visual acuity (BCVA) using the Snellen chart, subjective refraction, slit-lamp biomicroscopy, and a dilated fundus examination.

Regarding multimodal imaging, fundus photography, infrared fundus imaging, and fundus autofluorescence (FAF) using Optos, California (Optos, Malborough, MA, USA) or Topcon 3D OCT- 2000FA plus (Oakland, NJ, USA), spectral domain optical coherence tomography (SD-OCT) of the macula using Heidelberg Spectralis OCT (Heidelberg Engineering GmbH; Dossenheim, Germany) or Topcon 3D OCT- 2000FA plus (Oakland, NJ, USA) were obtained.

Additionally, full field and pattern electroretinogram (ffERG and PERG) were recorded using the RETIscan (Version 6.14.1.7, Roland Consult; Stasche & Finger GmbH, Brandenburg an der Havel, Germany) or LKC UTAS SunBurst (LKC Technologies, Inc., Gaithersburg, MD) according to the International Society for Clinical Electrophysiology (ISCEV) standards [27].

### Molecular analysis

Five milliliters of venous blood were withdrawn from each patient. DNA extraction, PCR for amplification, and Sanger sequencing of the 6 coding exons of the *RS1* gene were followed by alignment of the query sequence against the reference sequence. Different in-silico functional analysis tools were used to predict the pathogenicity of unreported variants. Family segregation was carried out for parents of patients showing a pathogenic variant in the *RS1* gene. Whole exome sequencing (WES) was undertaken for patients with no variant in the *RS1* gene. Bioinformatic analysis of the output data was done.

Array CGH was performed for patient 3 (P3), as well as both of her parents. The Cytoscan HD array kit (Affymetrix, Santa Clara, CA, USA; Thermo Fisher Scientific, Waltham, MA, USA) was used, which incorporates both copy number variation (CNV) probes and single-nucleotide polymorphism (SNP) probes. It is used to detect the presence of uniparental disomy (UPD). The procedure was performed according to the manufacturer’s instructions.

## Results

### Clinical results

Five patients (three females and two males) from three unrelated consanguineous families were included in this study. Age at first visit ranged from 15 to 37 years with a mean of 21.8 years. The main complaint was defective vision in all patients. None of the patients had any history of previous ocular surgeries, systemic conditions, or medications, specifically niacin or taxane agents.

### Clinical Findings

**Fig. 1:**
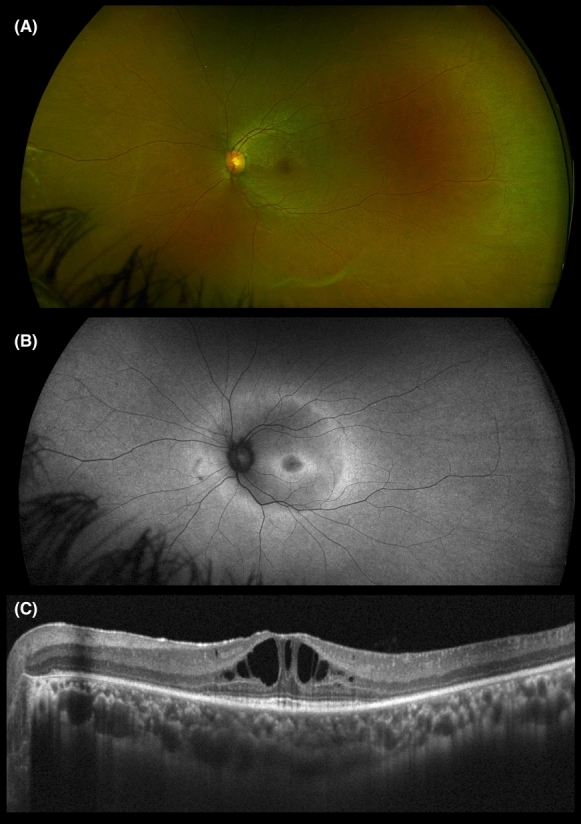
Multimodal imaging of patient 1 (P1). **A** Ultra-wide field pseudocolor fundus image of the left eye demonstrating radial spoke wheel appearance of fovea with peripheral changes inferiorly. **B** Ultra-wide field green light short-wavelength fundus autofluorescence image of the left eye demonstrating double ring hyperautofluorescence. **C** OCT of the macula showing foveoschisis at the level of outer plexiform layer (OPL)/Henle fiber layer (HFL) and outer retinal loss in the parafoveal region

**Fig. 2:**
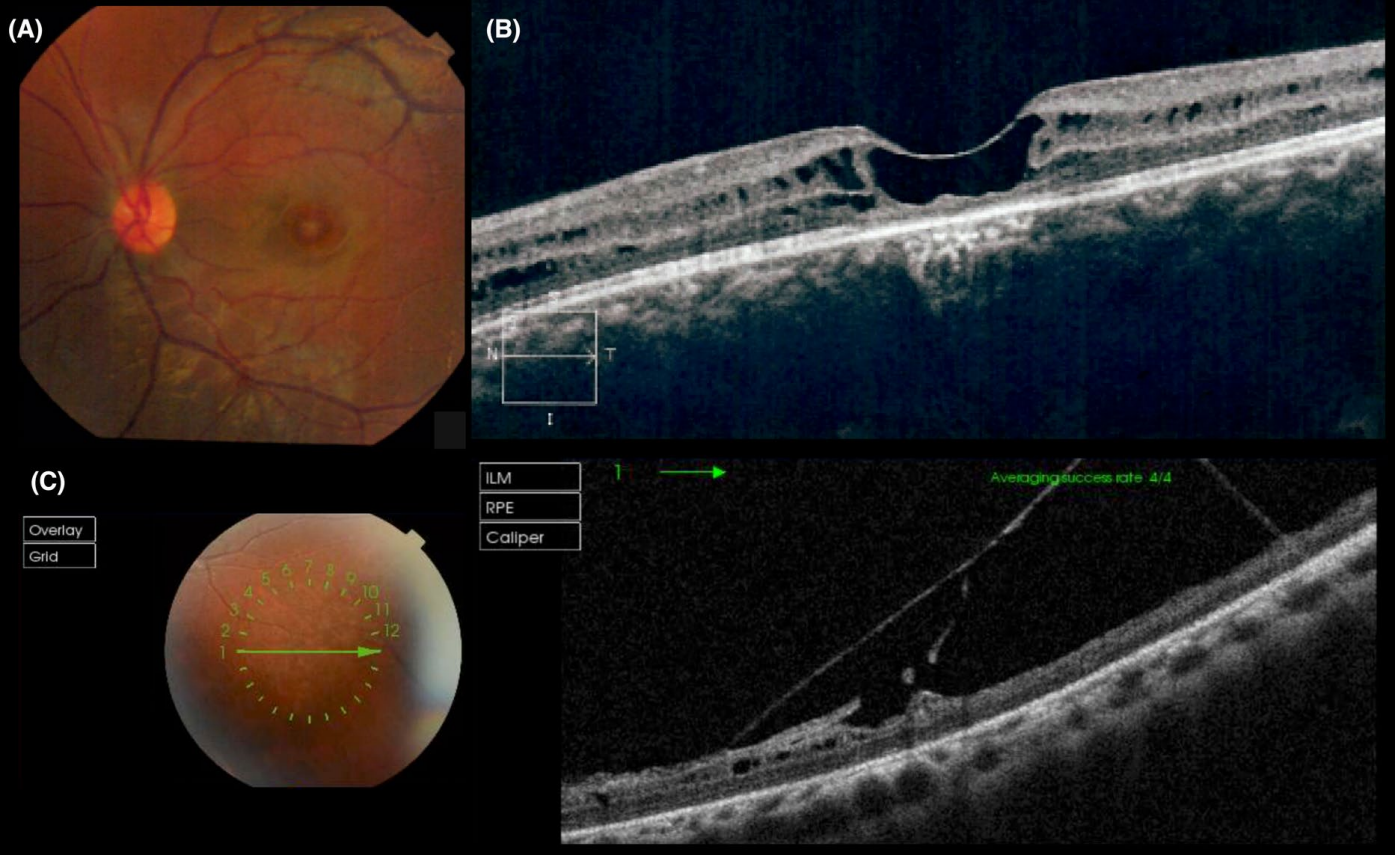
Fundus color images and OCT images of patient 2 (P2). **A** Color fundus photograph of the left eye showing classic spoke wheel pattern in the macular area. **B** OCT image of the macula of the left eye showing foveoschisis. **C** OCT image of the peripheral retina of the left eye depicting vitreoretinal traction

**Fig. 3:**
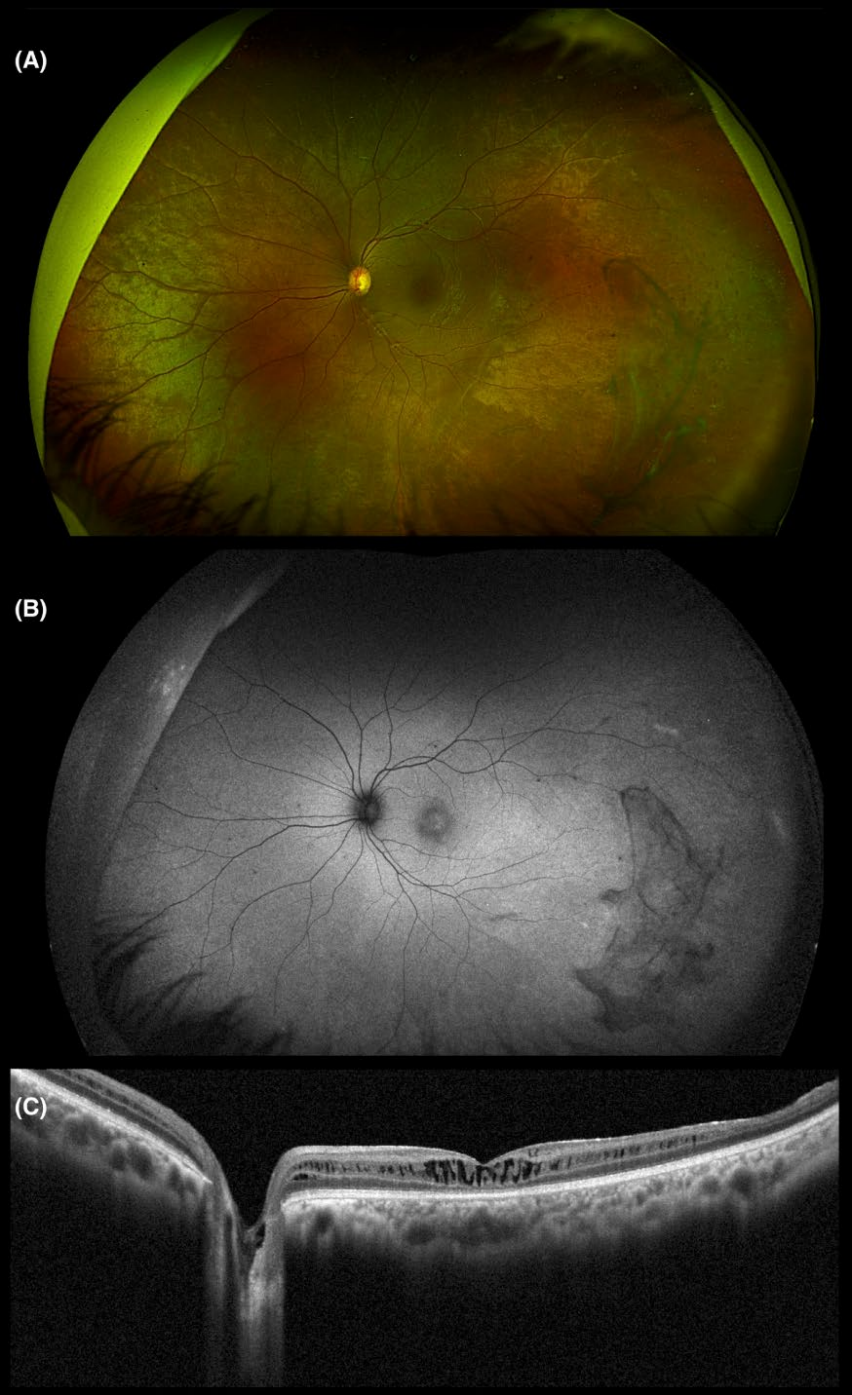
Multimodal imaging of patient 3 (P3). **A** Ultra-wide field pseudocolor fundus image of the left eye demonstrating radial spoke wheel appearance of fovea with peripheral changes inferotemporally along with vitreous veils. **B** Ultra-wide field green light short-wavelength fundus autofluorescence image of the left eye demonstrating an increased hyperautofluorescence signal. **C** OCT of the macula showing foveoschisis extending to the optic disc

Table 1 summarizes the clinical data of patients examined. On fundoscopy and on SD-OCT, all eyes of the studied patients exhibited foveoschisis (Fig. 1A, C, 2, 3A, C) (Supplementary Fig. 2A & C and 3A &C). Outer retinal loss in the parafoveal region was evident in patient 1 (P1) (Fig. 1A). Peripheral changes ranged from a tapetal reflex, peripheral retinoschisis, vitreous veils, to vitreoretinal traction (Fig. 1A, 2C, 3A). FAF assessment was available for all patients except patient 2 (P2) and revealed two distinct patterns. Two eyes showed an increased central signal, while four eyes exhibited a ring of increased signal. These findings were consistent with previously described FAF patterns (Fig. 3B) (Supplementary Fig. 2B, 3B) [28]. Double-ring hyperautofluorescence was observed in both eyes of P1 (Fig. 1B).

**Table 1:** Detailed clinical characteristics of patients with foveoschisis

Patient ID	Eye	Age (1st visit)/Sex	Family ID	Affected family members	BCVA	Refraction	Peripheral Fundus findings	Pattern of FAF	*RS1* Gene
1	OD OS	37/F	1	No	20/20 20/80	−0.50/−0.25 × 170 + 0.25/−0.50 × 55	Peripheral retinoschisis	Double-Ring Hyper-autofluorescence	Negative
2	OD OS	15/F	2	No	20/40 20/40	+ 1.00/−2.75 × 180 + 2.50/−2.75 × 180	Peripheral retinoschisis, vitreoretinal traction	N/A	Negative
3	OD OS	18/F	3	Yes	20/40 20/80	−1.50/−1.00 × 130 −3.75/−1.00 × 70	Peripheral retinoschisis, vitreoretinal traction, tapetal reflex, vitreous veils	Increased central signal	c.127 C > T, p. Gln43*
4	OD OS	23/M	3	Yes	20/100 20/63	+ 3.50/−1.50 × 100 + 4.00/−2.00 × 90	Tapetal reflex	Ring of increased signal	c.127 C > T, p. Gln43*
5	OD OS	16/M	3	Yes	20/63 20/63	−0.50/−0.75 × 45 −0.50/−0.50 × 65	Peripheral retinoschisis	Ring of increased signal	c.127 C > T, p. Gln43*

BCVA, best-corrected visual acuity; F, female; FAF, fundus autofluorescence; M, Male; N/A, not available; OD, right eye; OS, left eye; *RS1*, retinoschisin gene

ffERG was performed in all patients where Table 2 details the ERG findings observed. Pattern ERG (PERG) showed a reduction in P50 wave amplitudes in all patients.

**Table 2:** Detailed electroretinogram characteristics observed on Full-field ERG

Patient ID	DA 3 and 10 a-wave	DA 3 and 10 b-wave	DA 3 and 10 b:a Ratio	DA 3 and 10 Waveform	LA 3.0 Responses	LA 30 Hz Flicker Responses
1	Borderline Subnormal	Subnormal	< 1.0 (0.85 and 0.7)	Electronegative	Normal	Normal
2	Normal	Subnormal	< 1.0 (0.8 and 0.74)	Electronegative	Borderline Subnormal	Borderline Subnormal
3	Normal	Subnormal	< 1.0 (0.7 and 0.68)	Electronegative	Abnormal	Delayed with reduced amplitudes
4	Normal	Subnormal	< 1.0 (0.86 and 0.68)	Electronegative	Abnormal	Delayed with reduced amplitudes
5	Normal	Subnormal	< 1.0 (0.76 and 0.66)	Electronegative	Abnormal	Delayed with reduced amplitudes

DA, dark-adapted; LA, light-adapted; PERG, pattern electroretinogram

### Molecular results

**Fig. 4:**
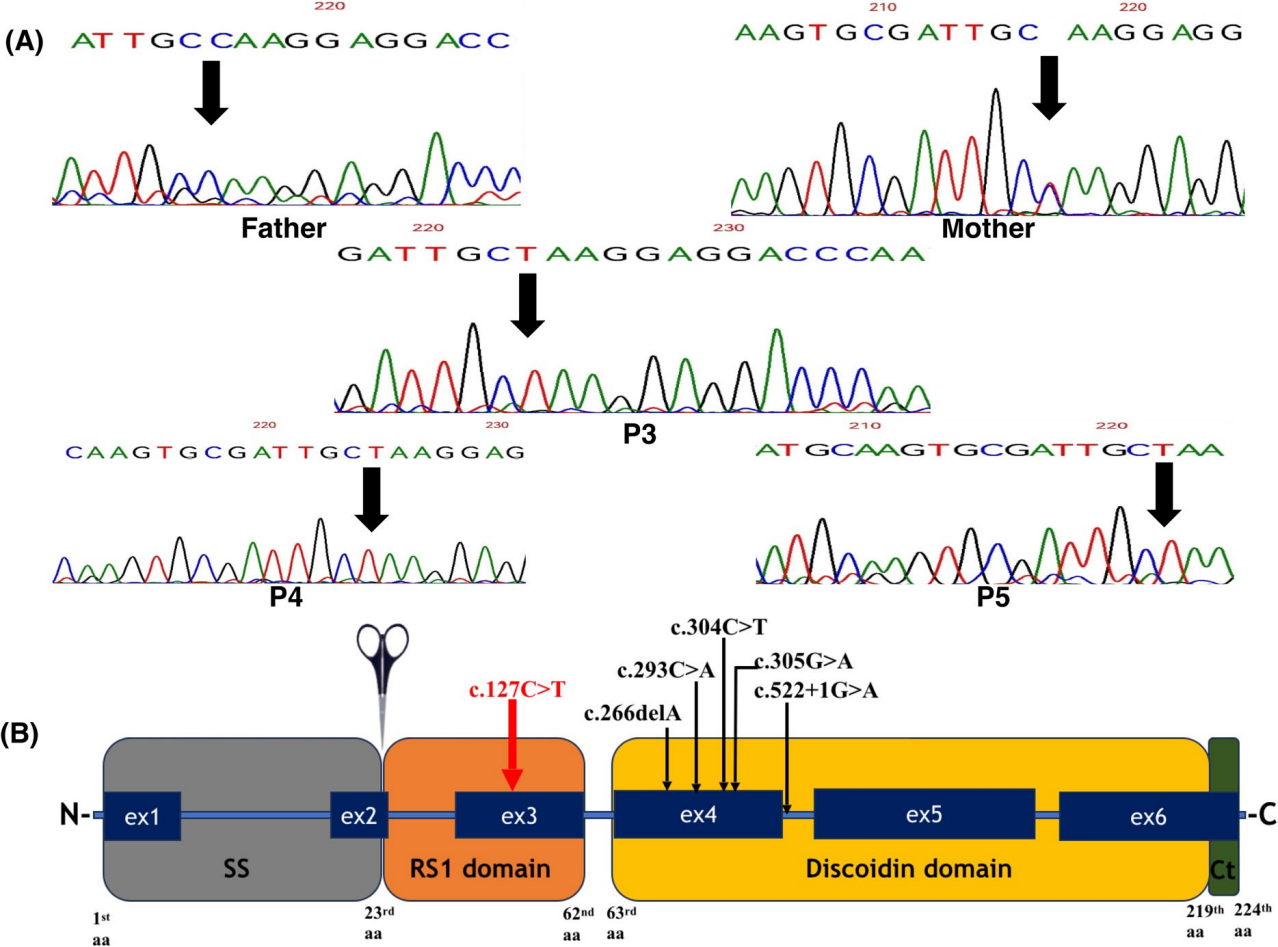
**A** Chromatograms of members of family 3 (F3) showing the detected novel variant in *RS1* gene (NM_000330.4: c. 127C > T, p. Gln43*) in a heterozygous form in the mother, absent in the father, in a homozygous form in P3 and in a hemizygous form in P4 and P5. **B** Illustrative figure of the *RS1* gene showing the cleavable N- terminal signal sequence (SS), RS1 domain, Discoidin domain, and C-terminal (Ct). The figure displays the variants detected in females throughout literature clustered in the Discoidin domain denoted in black and the novel variant detected in this study, located in the RS1 domain, denoted in red

Sanger sequencing revealed a normal sequence of the *RS1* gene in P1 and P2. WES was done to ensure the absence of variants in other genes that may present with foveoschisis, and it proved to be negative for both patients. Alternatively, it detected a novel variant (NM_000330.4 (RS1); c.127 C > T, p. Gln43*) in the three affected siblings (P3, P4, and P5) in family 3 (F3) (Supplementary Fig. 1). The variant was not previously detected in any population (gnomAD V4.1.0) as shown in Table 3. In silico prediction tools such as DANN, Bayesdel, Genocanyon, and Mutation Taster supported the deleteriousness of the variant. The variant was hemizygous in the two male siblings and homozygous in the female patient. Family segregation showed that the mother harbored the variant in a heterozygous state; it was not found in the father (Fig. 4A). The variant is located within the RS1 domain, as shown in Fig. 4B.

**Table 3:** Pathogenic variants detected in the *RS1* gene in female patients in the literature

Nucleotide change (genomic position)	Protein change	Variant Type	ACMG Classification (Criteria)	REVEL	Allele Frequency (gnomAD v4.1.0)	Splice Al	Zygosity	Postulated inheritance mechanism	References
c.127C > T (ChrX-18656710 G > A)	p. Gln43*	Nonsense	Likely pathogenic (PVS1, PM2)	N/A	Variant not found	N/A	Homozygous	Seg UPD	This study
c.266delA (ChrX-18647250 AT > A)	p. Tyr89Leufs*37	Frameshift	Likely pathogenic (PVS1, PM2)	N/A	Variant not found	N/A	Heterozygous	Skewed X-inactivation	Kirkby et al. [19]
c.293C > A (ChrX-18647224 G > T)	p. Ala98Glu	Missense	Pathogenic (PM1, PM2, PM3, PP1, PP2, PP3, PP5)	Deleterious (Moderate) (0.87)	Variant not found	N/A	Homozygous	Bi-allelic inheritance	Gliem et al. [29]
c.304C > T (ChrX-18647213 G > A)	p. Arg102Trp	Missense	Pathogenic (PM1, PM2, PM3, PM5, PP2, PP3, PP5)	Deleterious (Strong) (0.96)	0.000004955	N/A	Homozygous	Bi-allelic inheritance	Staffieri et al. [30]
c.305G > A (ChrX-18647212 C > T)	p. Arg102Gln	Missense	Pathogenic (PM1, PM2, PM3, PM5, PP2, PP3, PP5)	Deleterious (Strong) (0.98)	0.000008273	N/A	Heterozygous	Skewed X-inactivation	Saldana et al. [31]
c.522 + 1G > A (ChrX-18644429 C > T)	p.(?)	Splice donor	Pathogenic (PM2, PM3, PVS1, PP5)	N/A	Variant not found	Splice-Altering / strong (0.97)	Hemizygous	XO female (Turner’s syndrome)	Sato et al. [32]

REVEL is an ensemble way for predicting the pathogenicity of missense variants depending on 13 individual tools: MutPred, FATHMM, VEST, PolyPhen, SIFT, PROVEAN, MutationAssessor, MutationTaster, LRT, GERP, SiPhy, phyloP, and phastCons. The score ranges from 0 to 1 for an individual missense variant, where variants with a higher possibility of causing disease have higher scores. Splice AL predicts the incidence of splicing events with a score ranging from 0 to 1 where a higher score has a higher probability of being splice-altering.

*N/A* not available.

**Fig. 5:**
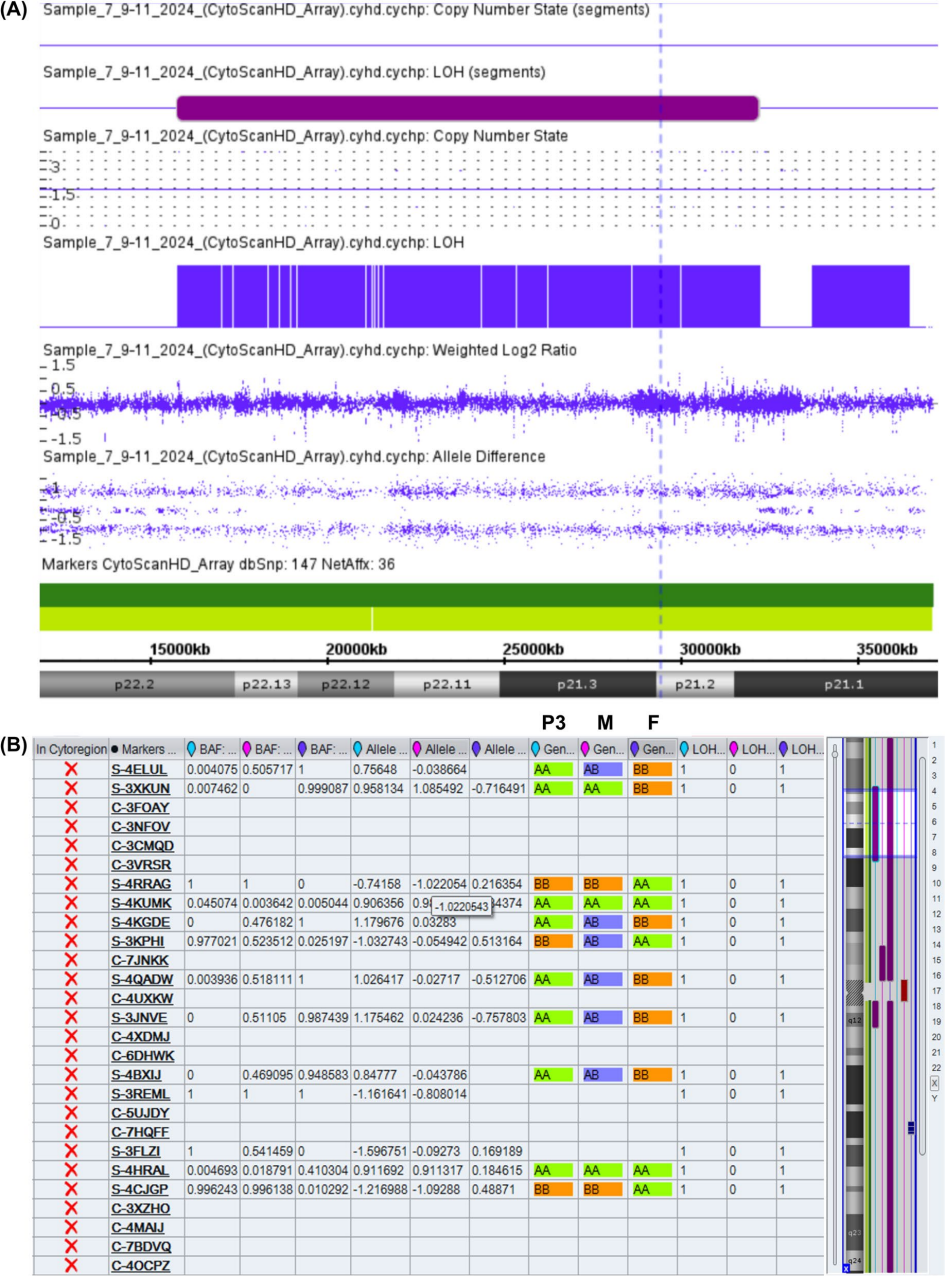
**A** SNP array results of P3. Chromosomal regions of uniparental isodisomy are shown as copy number neutral (= 2) loss of heterozygosity (LOH), and these regions do not include heterozygous SNP probe signals (AB). Segmental UPiD of chromosome X is seen in patient 3 which extended for 16 Mb. **B** SNPs for trios, P3: Patient 3, M: mother and F: father. SNP analysis of P3 revealed loss of heterozygosity (LOH) spanning 16 Mb segment [arr[GRCh38] Xp22.2p21.1(15780080_32210397) hmz] involving the *RS1* gene. SNP probes covering Xp22.2p21.1 for determining the allelic inheritance pattern demonstrated homozygosity (AA or BB) in P3 resembling inheritance pattern on one maternal allele and different from the paternal allele indicating that both copies of this segment originated from the same maternal chromosome (i.e., segmental UPiD of Xp22.2p21.1) and duplicated via UPiD

Further cytogenetic testing was conducted to determine the mode of inheritance. Both the father and P3 underwent karyotyping to detect any numerical or structural abnormality. Both had a normal karyotype. Trio SNP analysis [father, mother, and daughter (P3)] was performed to detect UPD and its type (isodisomy or heterodisomy) through the detection of allele inheritance patterns of specific SNP markers at multiple SNP positions on the X chromosome. SNP analysis for P3 revealed several long stretches of UPD loss of heterozygosity (LOH) across many chromosomes, indicating paternal consanguinity. LOH is the loss of one parental allele at a specific genomic locus, resulting in the conversion of a heterozygous genotype (two different alleles) to a homozygous or hemizygous state (where only one allele is present). This LOH may arise from deletions or from copy-neutral events, such as UPD, in which both chromosomal copies are inherited from the same parent. A 16 Mb segment of LOH was detected on the short arm of the X chromosome, arr[GRCh38] Xp22.2p21.1(15780080_32210397) × 2 hmz mat (Fig. 5A). It also revealed segmental maternal UPiD where homozygosity across different SNP loci on the X chromosome could be detected in P3 resembling one allele pattern of the mother, thereby confirming that a segment encompassing the *RS1* gene on both X chromosomes of P3 to which those SNP markers belong is exclusively inherited from the mother. Alternatively, the father showed a different allele inheritance pattern at those markers (Fig. 5B).

## Discussion

Foveoschisis refers to retinal splitting involving the fovea. Given the wide spectrum of potential underlying conditions, establishing an accurate diagnosis is essential, as each entity possesses a distinct natural history and functional outcome, with direct implications for patient counseling.

SNIFR is a rare cause of foveoschisis with a favorable functional outcome and is considered a diagnosis of exclusion after ruling out other possible conditions. The condition was first described by Ober et al. in 2014, with a noted female predominance, and may present either unilaterally or bilaterally [17].

Molecular analysis of the *RS1* gene can help in proper differentiation between XLRS and SNIFR. About 441 variants have been reported in the *RS1* gene so far [33]. Variants affecting the discoidin domain (encoded by exons 4–6) and the RS1 domain are the most frequently reported. Mutations within the signal sequence (SS) and C-terminal regions are less common. Approximately half of the identified variants are either nonsense or missense mutations. The high prevalence of variants in the discoidin domain may be due to its highly conserved nature across all proteins containing this domain. Furthermore, the discoidin domain includes cysteine residues critical for forming the disulfide bonds necessary for the secretion of retinoschisin from retinal cells. This secretion process yields a disulfide-linked homo-octameric complex, which is essential for maintaining retinal integrity through its binding to the surface of photoreceptors and bipolar cells [1].

Variants in the *RS1* gene might hinder retinoschisin secretion from the ER, its octamerisation, or its function [1]. These mechanisms are predicted based on the variant’s position within the protein. No genotype–phenotype correlation could be established in XLRS patients, which might be attributed to the presence of modifier genes or environmental influences [18].

We have concluded that P1 might be a moleculary undiagnosed IRD whose variant has not yet been found. Molecularly undiagnosed IRD cases primarily result from limitations in conventional genetic testing methodologies, which predominantly target coding exons and canonical splice sites, thereby missing deep intronic mutations and regulatory region alterations. Additionally, novel disease genes further contribute to unresolved cases.

On the other hand, we concluded that P2 had a diagnosis of SNIFR as she was not myopic, her optic discs showed no evidence of pits, there was no evidence of VMT, and her systemic and drug histories were unremarkable. Moreover, we were able to exclude *RS1* variants via Sanger sequencing and *NR2E3* or *CRB1* variants via WES. However, in view of her grossly abnormal ERG findings an alternative diagnosis of cone-rod dystrophy with associated inner retinal dysfunction may be considered.

SNIFR can be associated with peripheral retinal changes [34, 35]. Our case exhibited peripheral retinoschisis and vitreoretinal traction. Regarding the ERG changes, SNIFR has been reported to have controversial changes. Normal ffERG was reported by Barbano et al. [36], supranormal amplitudes were reported by Dolz-Marco [37], and diminished scotopic and photopic responses were also reported [38, 39]. Our patient (P2) demonstrated reduced amplitudes of b-wave with b:a ratio less than 1, resulting in an electronegative waveform, similar to that seen with XLRS, which further complicates the clinical assessment and highlights the need for genetic testing for differentiation. This was previously reported in only two cases [40, 41].

When we assessed the patterns of FAF in our patients, we found a novel pattern of a double-ring of hyperautofluorescence in P1 with a probable diagnosis of non-specified IRD. This pattern has been previously described in *USH2A*-retinopathy [42, 43], *CFAP410*-related retinopathy [44], and *NR2E3*-associated autosomal dominant retinopathy [45].

The third family included 3 affected siblings with unaffected consanguineous parents. The 3 siblings carried a novel nonsense variant (NM_000330.4 (RS1): c.127 C > T, p. Gln43*). This variant is a stop gain variant producing a truncated protein (43aa) with possible nonsense-mediated decay of the produced mRNA, thus exerting a loss-of-function (LOF) effect. To date, 79 LOF variants have been reported downstream and 19 upstream of this variant. Alternatively, variants resulting in a longer protein can also be pathogenic, for example c.639delG variant, which was first detected in a Colombian family [23].

XLRS which is a close differential that resembles SNIFR phenotypically, where XLRS is almost exclusively seen in young males who harbour *RS1* gene variants, with the condition rarely affecting females. Only 14 of the 31 females described in 16 studies in the literature have undergone molecular analysis [19]. Our study is the first to report an RS1 domain variant in a female patient; all previously identified variants were located in exon 4 and the fifth intervening sequence [19, 29–32].

Several genetic mechanisms may explain why a female is affected by XLRS. One possibility is that the female has homozygous or compound heterozygous variants, which can occur in the offspring of consanguineous marriages or from the union of carrier parents from unrelated families. Additionally, skewed inactivation of the X chromosome carrying the normal allele has been proposed as an explanation, a hypothesis supported by several studies, although it is considered rare [19, 31]. Another potential scenario is germline mosaicism, in which a variant is present in some of the parents' germline cells but not in their somatic cells. In this case, the variant can be passed to the offspring even if it's not detected through standard genetic testing. Other possibilities mentioned in the literature include an autosomal dominant mode of inheritance [46] and, in a very rare instance, the presence of Turner's syndrome (XO female) [47] or paternal uniparental disomy (UPD) of the X chromosome. To the best of our knowledge, no XLRS female cases with UPD have been reported in the literature so far as shown in Table 2.

In our case, Trio SNP array proved that the maternal segmental uniparental isodisomy (seg UpiD) has taken place in a region about 16 MB including the *RS1* gene on the X chromosome where a homozygous allelic pattern for P3 at this region resembling the pattern on one maternal allele was detected with a different paternal allelic pattern at that particular region, hence explaining the biallelic inheritance from the maternal side. UPD occurs when two copies of a whole chromosome are derived from the same parent with a prevalence rate of 1:2000 births and a 75% probability of maternal UPD [48]. Maternal heterodisomy, where both maternal allele sets are inherited, is more common than maternal isodisomy (duplicated single set of alleles), whereas seg UPiD is the rarest form [49]. Seg UPiD and whole chromosome UPD originate from different mechanisms. Whole chromosome UPD is usually due to a nondisjunction event where a pair of homologous chromosomes has failed to separate at anaphase, causing a pair of chromosomes to pass to one daughter cell instead of a single chromosome. Alternatively, Seg UPiD is a consequence of somatic cell recombination between chromatids followed by segregation of the chromatids with identical allelic segments [48]. This interpretation is consistent with P3, in whom two identical maternal X-chromosome segments, each harboring the *RS1* mutation, resulted in homozygosity despite the absence of the variant in the father, thereby excluding paternal germline mosaicism.

In conclusion, we recommend that molecular testing is considered for the assessment of females presenting with foveoschisis. Moreover, we expand the molecular spectrum of XLRS with a novel stop gain LOF variant and the first in the RS1 domain in a female patient. We introduce, with evidence, the first female XLRS patient with non-Mendelian inheritance due to maternal Seg UPiD.

## Supplementary Information

Below is the link to the electronic supplementary material.

Supplementary Fig.1: Three-generation pedigree chart of the family to whom patient 3 (P3), 4 (P4) and 5 (P5) belong. The pedigree highlights the consanguineous nature of the family. Note the affected paternal grandfather

Supplementary Fig. 2: Multimodal imaging of patient 4 (P4). (A) Ultra-wide field pseudocolor fundus image of the left eye demonstrating radial spoke wheel appearance of fovea with a peripheral tapetal reflex. (B) Ultra-wide field green light short-wavelength fundus autofluorescence image of the left eye demonstrating a ring of increased hyperautofluorescence signal. (C) OCT of the macula showing marked foveoschisis

Supplementary Fig. 3: Multimodal imaging of patient 5 (P5). (A) Ultra-wide field pseudocolor fundus image of the left eye demonstrating radial spoke wheel appearance of the fovea with peripheral changes inferotemporally. (B) Ultra-wide field green light short-wavelength fundus autofluorescence image of the left eye demonstrating a ring of increased hyperautofluorescence signal. (C) OCT of the macula showing foveoschisis extending to the optic disc

Supplementary Fig. 4: Full-field Electroretinogram of the three female patients, patient 1 (P1), patient 2 (P2) and patient 3 (P3)

## Data Availability

The authors confirm that the data supporting the findings of this study are available within the article [and/or] its supplementary materials.

## Declarations

### Statement on the welfare of animals

No animals were used in the study, and no procedures were performed on animals. As such, ethical approval for animal research was not required.
