# Supplementary figures and images for: Molecular analysis of foveoschisis in females reveals a novel case of segmental uniparental disomy in X-linked retinoschisis
Source: Doc Ophthalmol. 2025 Oct 22;152(2):145–58. doi: 10.1007/s10633-025-10053-y (PMC13083353; doi:10.1007/s10633-025-10053-y)

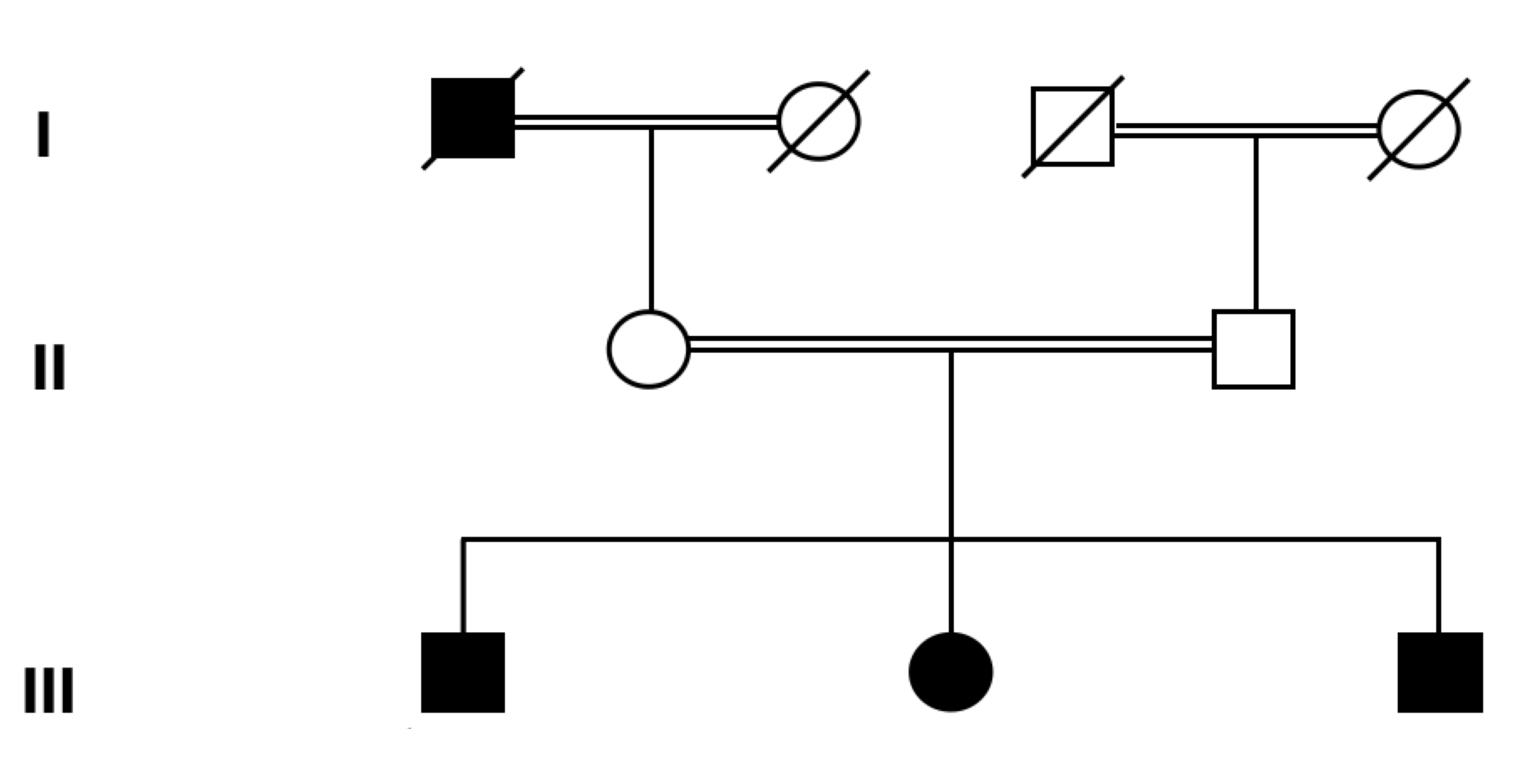

Supplement: Supplementary file 1 — Supplementary Fig.1: Three-generation pedigree chart of the family to whom patient 3 (P3), 4 (P4) and 5 (P5) belong. The pedigree highlights the consanguineous nature of the family. Note the affected paternal grandfather [file 10633_2025_10053_MOESM1_ESM.jpg]

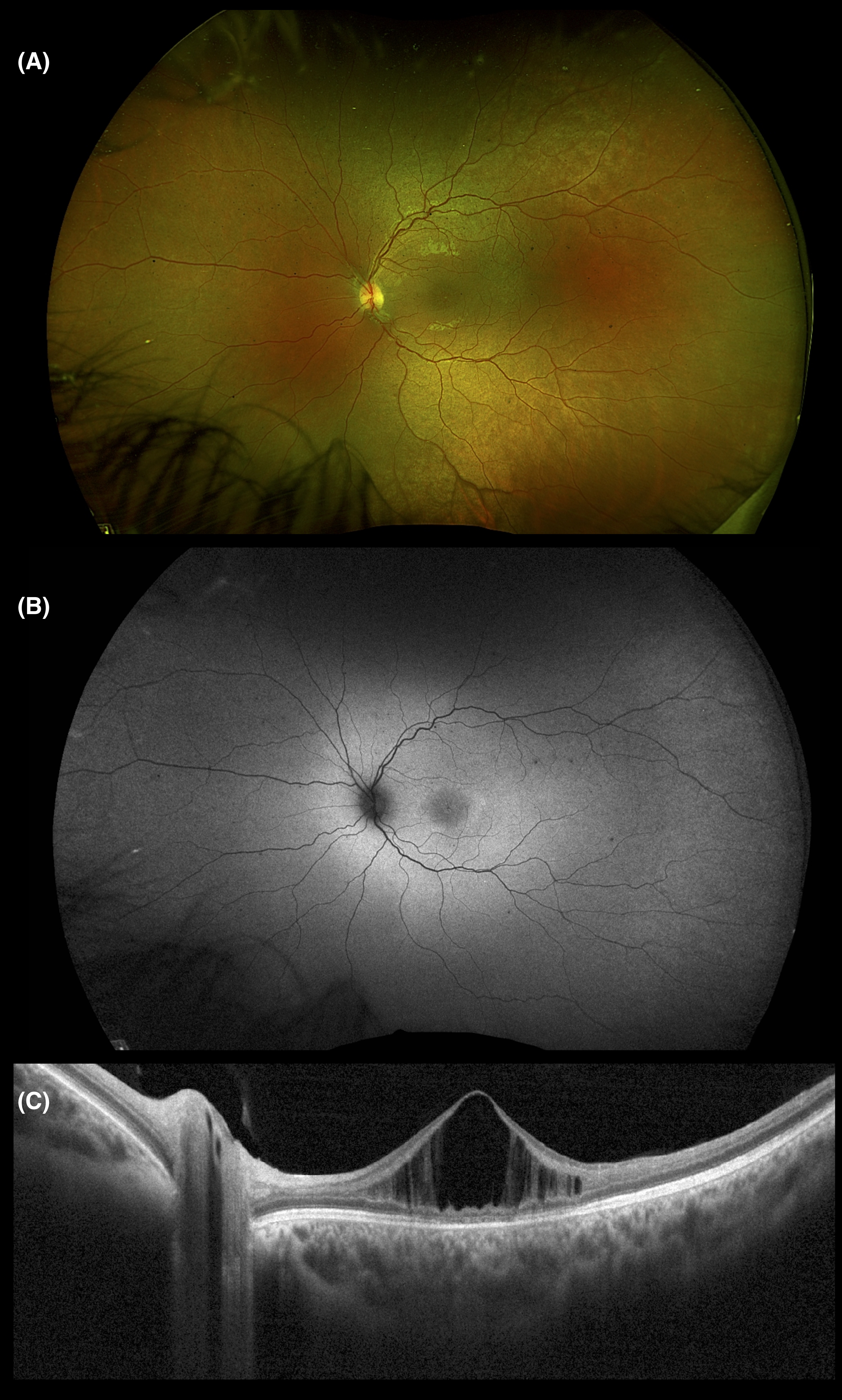

Supplement: Supplementary file 2 — Supplementary Fig. 2: Multimodal imaging of patient 4 (P4). (A) Ultra-wide field pseudocolor fundus image of the left eye demonstrating radial spoke wheel appearance of fovea with a peripheral tapetal reflex. (B) Ultra-wide field green light short-wavelength fundus autofluorescence image of the left eye demonstrating a ring of increased hyperautofluorescence signal. (C) OCT of the macula showing marked foveoschisis [file 10633_2025_10053_MOESM2_ESM.jpg]

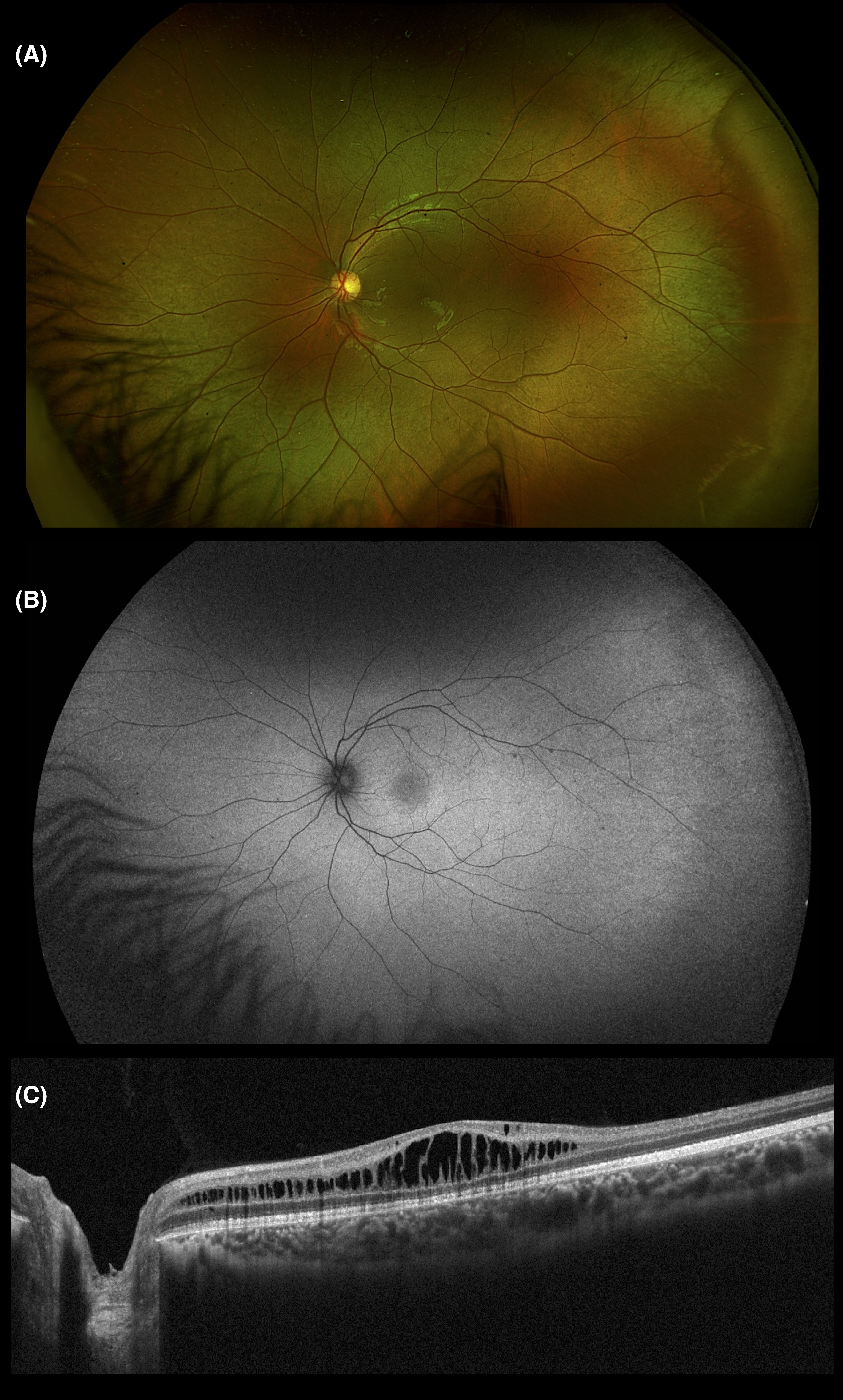

Supplement: Supplementary file 3 — Supplementary Fig. 3: Multimodal imaging of patient 5 (P5). (A) Ultra-wide field pseudocolor fundus image of the left eye demonstrating radial spoke wheel appearance of the fovea with peripheral changes inferotemporally. (B) Ultra-wide field green light short-wavelength fundus autofluorescence image of the left eye demonstrating a ring of increased hyperautofluorescence signal. (C) OCT of the macula showing foveoschisis extending to the optic disc [file 10633_2025_10053_MOESM3_ESM.jpg]

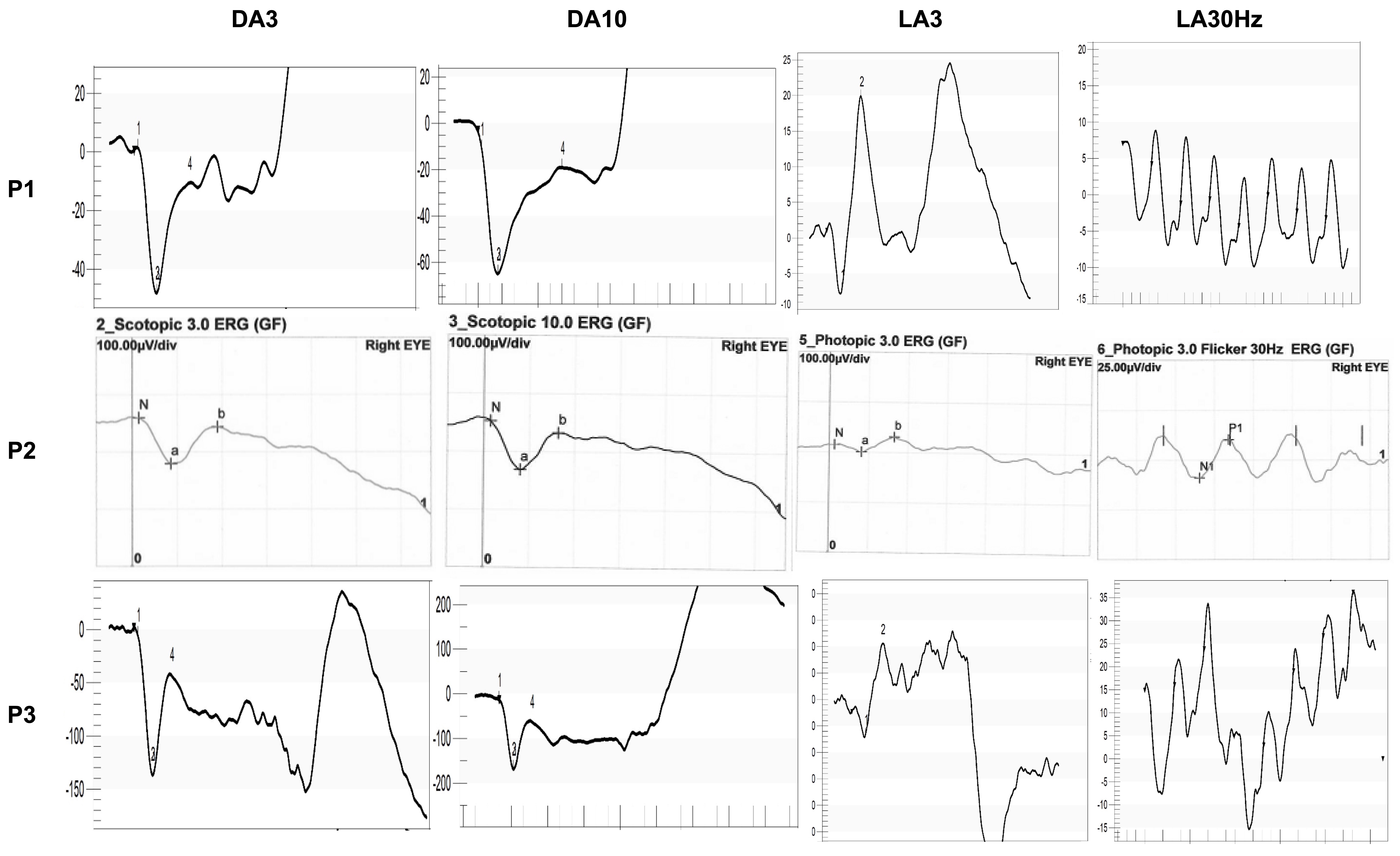

Supplement: Supplementary file 4 — Supplementary Fig. 4: Full-field Electroretinogram of the three female patients, patient 1 (P1), patient 2 (P2) and patient 3 (P3) [file 10633_2025_10053_MOESM4_ESM.jpg]
